# Seagrass Proliferation Precedes Mortality during Hypo-Salinity Events: A Stress-Induced Morphometric Response

**DOI:** 10.1371/journal.pone.0094014

**Published:** 2014-04-04

**Authors:** Catherine J. Collier, Cecilia Villacorta-Rath, Kor-jent van Dijk, Miwa Takahashi, Michelle Waycott

**Affiliations:** 1 School of Marine and Tropical Biology, James Cook University, Townsville, Australia; 2 School of Earth and Environmental Science, Australian Centre for Evolutionary Biology and Biodiversity, University of Adelaide, Adelaide, Australia; Dauphin Island Sea Lab, United States of America

## Abstract

Halophytes, such as seagrasses, predominantly form habitats in coastal and estuarine areas. These habitats can be seasonally exposed to hypo-salinity events during watershed runoff exposing them to dramatic salinity shifts and osmotic shock. The manifestation of this osmotic shock on seagrass morphology and phenology was tested in three Indo-Pacific seagrass species, *Halophila ovalis*, *Halodule uninervis* and *Zostera muelleri*, to hypo-salinity ranging from 3 to 36 PSU at 3 PSU increments for 10 weeks. All three species had broad salinity tolerance but demonstrated a moderate hypo-salinity stress response – analogous to a stress induced morphometric response (SIMR). Shoot proliferation occurred at salinities <30 PSU, with the largest increases, up to 400% increase in shoot density, occurring at the sub-lethal salinities <15 PSU, with the specific salinity associated with peak shoot density being variable among species. Resources were not diverted away from leaf growth or shoot development to support the new shoot production. However, at sub-lethal salinities where shoots proliferated, flowering was severely reduced for *H. ovalis*, the only species to flower during this experiment, demonstrating a diversion of resources away from sexual reproduction to support the investment in new shoots. This SIMR response preceded mortality, which occurred at 3 PSU for *H. ovalis* and 6 PSU for *H. uninervis*, while complete mortality was not reached for *Z. muelleri*. This is the first study to identify a SIMR in seagrasses, being detectable due to the fine resolution of salinity treatments tested. The detection of SIMR demonstrates the need for caution in interpreting in-situ changes in shoot density as shoot proliferation could be interpreted as a healthy or positive plant response to environmental conditions, when in fact it could signal pre-mortality stress.

## Introduction

Seagrasses are a group of angiosperms (flowering plants), within the monocotyledon order Alismatales [1], [2]. Seagrasses evolved along four separate lineages but are considered a single functional group because of similar adaptive traits, principally their tolerance to seawater salinities [1]. Their preferred salinity ranges from 20 practical salinity units (PSU) through to 42 PSU, except for *Ruppia* spp which frequently inhabit fresh water (0 PSU) [3].

Seagrasses predominantly occur in estuaries and coasts where salinity can be affected by watershed run-off leading to hypo-saline conditions [4], or it can become hyper-saline in shallow embayments with high rates of evaporation [5], [6] and at sites of desalinisation discharge [7]. In tropical and monsoonal climates, wet season depressions in salinity can reach 0 PSU during extreme runoff events [8]. Run-off can be associated with widespread declines in seagrass abundance, with significant consequences for the broader ecosystem [9], [10]. A number of studies have described the effects of hypo-salinity on northern hemisphere seagrass species in Europe and the USA [4], [11]–[13]; however, sensitivity to hypo-salinity is not known for most Indo-Pacific seagrass species. Furthermore, previous seagrass studies, with some exceptions [13], have lacked the treatment and temporal resolution to determine hypo-salinity thresholds whereby extreme mortality occurs. Without these thresholds it is difficult to determine what role hypo-salinity stress has during mortality associated with watershed run-off.

Salinity affects water uptake, plant water potential and cellular ion concentrations, and when plants become salinity-stressed there are damaging consequences for cellular integrity, biochemical processes and ultimately, plant fitness [14], [15]. Seagrasses are halophytes, that is, they maintain high intracellular osmotic potentials in saline environments, by ion sequestration and the generation of osmotically-active solutes [3], [15]. These osmolytes enable seagrasses to exclude Na+ and Cl- ions even at very high concentrations [3], [15]. Exceedance of optimum salinity and disruption of cellular processes affects photosynthetic efficiency and leads to reduced growth rates and morphological changes and eventual mortality [11], [15]–[19].

The duration of exposure affects the level of impact on plant fitness and seagrasses may recover following brief levels of exposure to salinity stress but may fail to recover after prolonged stress [15], [19]. Furthermore, the rate of salinity change affects plant health, with incremental salinity change increasing plant survivorship [11], [12], [19]. This is an important consideration when testing plant survivorship as hypo-salinity changes are rarely sudden – even though experimental approaches frequently assume so – but rather they occur gradually as flood waters mix with saline waters [5], [12].

We tested response to hypo-salinity of three seagrass species that inhabit estuarine and coastal environments where marine salinity is typical, but seasonal hypo-salinity events are common [8]. We mimicked the gradual reduction in salinity that would be expected as flood waters emerge from watersheds and flood into estuaries and coasts. This detailed approach revealed not just broad salinity tolerance but also a stress-induced morphogenic response (SIMR) [20]–[22] in which shoot proliferation occurred – a stress response not previously reported for seagrass.

## Materials and Methods

All plants were collected under permit MTB41, issued by the School of Marine and Tropical Biology, at James Cook University, in accordance with low impact research guidelines in the Great Barrier Reef Marine Park.

### Experimental conditions

**Figure 1:**
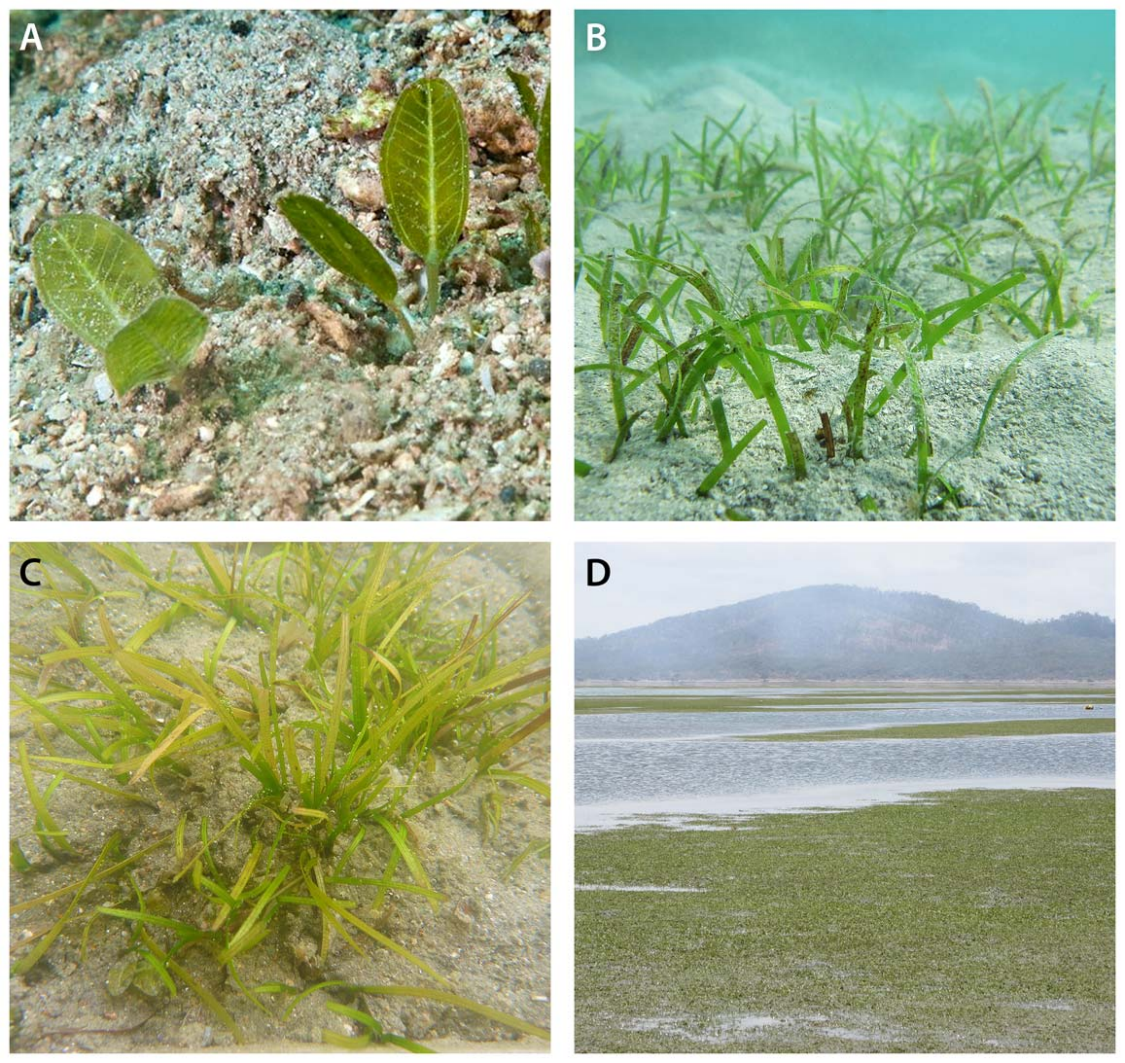
*Halophila ovalis* (A), *Halodule uninervis* (B), *Zostera muelleri* in the experimental units after 3 weeks exposure to 9 PSU (C) and a *Zostera muelleri* meadow in Gladstone Harbour, Australia where experimental plants were collected (D).

Hypo-salinity exposure experiments were conducted on three species of seagrass, which are ubiquitous throughout the Indo-Pacific, except *Zostera muelleri* Irmisch ex Ascherson, which is widespread in Australia and New Zealand only (Fig 1). *Halodule uninervis* (Forsskål) Ascherson is a tropical species that occurs throughout the Indo-West Pacific in coastal and reef habitats, while *Halophila ovalis* R. Brown is one of the most broadly distributed seagrass species occurring throughout the Indo-West Pacific, including temperate regions, and can be found in estuarine, reef and deepwater habitats [23]. Their habitats are periodically exposed to flood plumes of reduced salinity [8]. Both *Z. muelleri* and *H. uninervis* are species with linear leaf blades (blady), whereas *H. ovalis* has pairs of ovate leaves arising from the rhizome on petioles (Fig 1).

*Zostera muelleri* plants were collected from Pelican Banks, Gladstone (23°45.895′S, 151°18.244′E) during low tide three months before the experiments started. The plants were collected using a 10 cm corer, with sediment and rhizome and roots collected intact. The cores were placed in plastic-lined pots, the plastic bag sealed over the top of the seagrass with 2–3 cm of water during transport to the experimental facility. *Halodule uninervis* and *H. ovalis* plants were collected from Cockle Bay, Magnetic Island (19°10.612S, 146°49.737E) using the same technique two months prior to the experiments. The plants were kept in 1000L aquaria at the Aquaculture facility in James Cook University on a closed circulation system in seawater piped from Bowling Green Bay seawater intake under a 30% light-reducing roof.

The experiment consisted of 12 salinity treatments, starting from 3 PSU and increasing by 3 PSU to 36 PSU (approximate marine seawater). Salinity treatments were obtained by diluting the seawater with de-chlorinated freshwater. Every salinity treatment consisted of four replicate tanks (65L KiTab clear plastic containers) with one pot of each species per tank (i.e. n = 4). All treatments started at 36 PSU and salinity was reduced by 25% each day over four days to the target treatment salinity to mimic the more gradual decline in salinities that occur during run-off events and to minimize potential impacts from shock osmotic changes. Throughout the experiment, salinity was measured every 1 to 3 days using a digital salinity/conductivity/temperature meter (YSI, model 63) and salinity was adjusted when necessary to maintain salinity within 0.5 PSU of target salinity. Plant responses to these salinities were monitored for 10 weeks. Previous salinity studies indicate that seagrass changes settle down by this time [5], [12], and furthermore, this experimental duration is approximately equal to or more likely exceeds the length of individual hypo-salinity events in the region.

**Figure 2:**
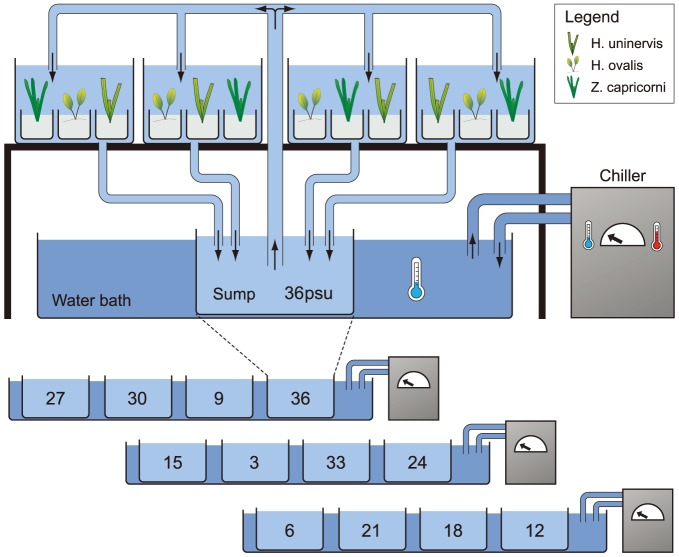
Experimental set-up showing three chilled water baths each with four randomly allocated sumps immersed within them. Each sump contained one of the 12 salinity treatments. Water was piped from the sump to the four replicate tanks and back again on closed-circulation.

The experiments were conducted outdoors during summer/autumn months (February to April) when high ambient temperatures occur, thus chilling units were installed to moderate temperature fluctuations within the treatment tanks throughout the experiment. There were three chilled freshwater baths (1000L tanks) that were cooled using external water chillers. Each of the 12 salinity treatments had one 60L sump (60L plastic bin) that was placed randomly in one of the 3 chilling baths, each bath containing 4 sumps (Fig 2). The chilled baths with sumps were held underneath tables that held the experimental tanks. From each sump, water with corresponding salinity was pumped into four replicate tanks resulting in a total of 48 tanks (4 replicate tanks ×12 sumps/salinity treatments  = 48 tanks in total). Each tank contained one pot of each of the three species (48 tanks ×3 species/pots  =  144 pots). Temperature was recorded every 30 mins using iBCod 22L model of iBTag in six randomly selected tanks for the duration of the experiment. Water temperature was 26°C on average and ranged from 22°C to 34°C reaching these temperature extremes for short periods (1–2 h) on some days. Nitrogen (N) as NH_4_Cl and phosphate (P) as KH_2_PO_4_ were added to the water column at very low concentrations to increase concentrations within each system by 0.05 μMol of P and 1.0 μMol of N every 2 weeks. Nutrient concentration was measured after six weeks and was found to be 0.8 μMol (±0.2) NH_3_, 0.4 μMol (±0.1) NOx, and 0.2 μMol (±0.8) PO_4_. Average light intensity under the 30% light-reducing roof was 17 mol photons m^−2^ d^−1^ of Photosynthetically Active Radiation (PAR), measured with an Odyssey 2Pi quantum sensor (Dataflow, Odyssey photosynthetic recording system) recording every 30 mins throughout the experimental period. The tanks were periodically cleaned by syphoning out sediment and organic matter accumulating at the bottom of tanks and plants were inspected every week for signs of grazing by amphipods. Amphipods were removed to prevent an outbreak, which could lead to overgrazing of the plants. Although signs of grazing were observed at times, this cleaning regime was sufficient to avoid outbreaks.

### Plant growth and survival

The number of shoots in each pot for *Z. muelleri* and *H. uninervis*, or the number of leaf pairs for *H. ovalis* were counted prior to the experiment and then weekly during the first four weeks of the experiment and fortnightly from the sixth week up to and including the tenth week. Change in shoot density (ΔSht) was calculated as a percentage change in each week relative to pre-treatment for each individual replicate:
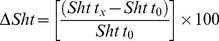
(1)where Δ*Sht* is change in shoot density, Sht *t_χ_* is shoot density at time x (weeks 1 through to 10) and Sht t_0_ is shoot density at week zero (pre-treatment).

Leaf morphometrics (width and height) of *Z. muelleri*, *H. uninervis* and *H. ovalis* and number of leaves per shoot for the two blady species were measured after 10 weeks at treatment salinity. These data were used to calculate foliar surface area (SA) as follows:
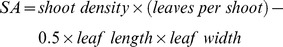
(2)for blady species (*H. uninervis and Z. muelleri*); and,
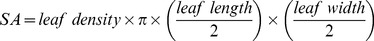
(3)for the ovate species *H. ovalis* where SA is the foliar leaf area (cm^2^), shoot density are leaves per experimental pot, leaves per shoot are the mean number of leaves (usually 1 to 4) per seagrass shoot and leaf length (cm) and leaf width (cm) of the youngest fully mature leaf. A half leaf was subtracted from the total number of leaves per shoot in calculating LA of blady species to account for one leaf on each shoot being in development and therefore not full sized [24].

*Halophila ovalis* was the only species to flower throughout the experimental period. Flowering had commenced prior to the initiation of the experiment and continued throughout. New leaf pairs are produced in *H. ovalis* every 3 or 4 days at experimental water temperatures of approximately 27–27°C [25] and *H. ovalis* typically had 4 to 5 leaf pairs per branch. Flowering is initiated in young leaf pairs, with more advanced reproductive structures away from the growing apex. Assuming a leaf pair production rate of 4 days, we conservatively assumed that all reproductive structures present after 4 weeks (28 d) were initiated under treatment conditions. We counted all reproductive structures (male and female flowers, as well as fruits) in each pot at weeks 4, 6, 8 and 10. We calculated reproductive potential – the highest number of reproductive structures occurring under treatment conditions as follows:
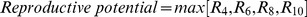
(4)where R_4_ is mean structures in week 4 of treatment salinity through to R_10_, which is mean structures in week 10. We also present the total number of reproductive structures against shoot density for each replicate.

Leaf growth rate was measured in week 10 on the two blady species (*Z. muelleri* and *H. uninervis*) using the leaf hole punch method [26]. Holes were punched using a hypodermic needle in the top of the sheath of each shoot, and after 5–7 days we measured the distance between the mark in the sheath and the mark on the leaves. We aimed to measure up to 10 shoots per replicate pot, though the actual number measured in each pot was variable depending on shoot density and visibility of marks.

### Statistical analyses

Shoot density data was analysed using a one-way repeated measures analysis of variance (RM ANOVA) with salinity as a fixed factor between-subjects effect and time (weeks) as the within-subjects effect. Data were first checked for homogeneity of variances using Levene's test, and transformed if failing this assumption of ANOVA. Transformation was not successful at improving variances at all times, typically one or two measuring times failed these tests (p<0.05) in which case the ANOVA was still performed on transformed data as the ANOVA is relatively robust to violations of assumptions in large experiments such as this; however, the significance level was set to 0.01 to minimize the risk of a Type II error [27]. Data were also checked for sphericity (correlations among time) and the degrees of freedom was adjusted using the Greenhouse-Geiser epsilon adjustment where necessary. Where a significant interaction between time and salinity was observed, post-hoc analyses to explore differences among treatments were performed for each measuring time separately. For single time data, single factor ANOVA's were performed with salinity as a fixed factor. Data were tested and treated as described above, and post-hoc analyses were conducted using S-N-K comparisons. All statistical analyses were performed using SPSS v20.0. Key statistical results are described in text with detailed statistical results in Tables.

## Results

### Shoot density

Initial shoot densities were on average 55 (± SE 8) leaf pairs for *H. ovalis*, 8 (± SE 1) shoots for *H. uninervis* and 30 (± SE 5) shoots for *Z. muelleri*. Changes in shoot density in response to hypo-salinity generally followed the same trends among species with a salinity response that was affected by time (Table 1); however, thresholds and response times were variable. The most notable difference among species was in their sensitivity at the lowest salinities; *H. ovalis* was the most sensitive, whereas *Z. muelleri* was the most tolerant of very low (3 and 6 PSU) salinities. Furthermore, *Z. muelleri* increased shoot density by the largest magnitude at low-mid salinities.

**Table 1:** Results of single factor repeated measures analysis of variance (RM-ANOVA) for change in shoot density at salinity treatments of 3 to 36 PSU in three seagrass species: *Halophila ovalis*, *Halodule uninervis* and *Zostera muelleri*.

		*H. ovalis*			*H. uninervis*			*Z. muelleri*	
	df	F	p	df	F	p	df	F	p
Within-subjects effects									
Time	2.580	9.432	**<0.001**	2.364	4.338	n.s.	3.131	78.380	**<0.001**
Time x salinity	28.384	21.949	**<0.001**	26.004	6.556	**<0.001**	34.445	11.848	**<0.001**
Between-subjects effects									
Salinity	11	44.170	**<0.001**	11	7.366	**<0.001**	11	19.531	**<0.001**
Transformations	4^th^Rt (x+101)			SqRt (x+101)			SqRt (x+101)		
Significance level (p)	0.01			0.01			0.01		

Transformations performed to meet assumptions of ANOVA and significance level used for interpretation of results are also indicated for each species.

**Figure 3:**
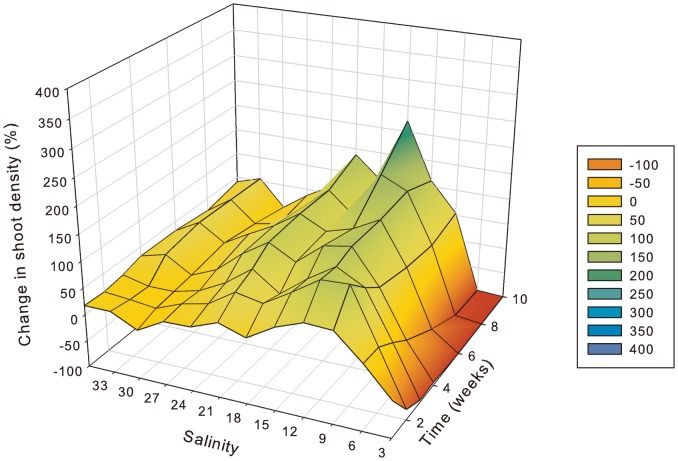
Change in *Halophila ovalis* shoot density relative to pre-treatment (week 0) (y-axis) as indicated by colour shading from 100% loss (red) through to 400% increase (blue), at salinities 3 to 36 PSU (x-axis) after 1 through to 10 weeks of exposure to treatment salinity (z-axis). n = 4.

More specifically, in *H. ovalis* leaf pair density had declined after a one-week exposure to 3 PSU (Fig 3) and after 2 weeks it was significantly (p<0.01) lower than all other salinity treatments (Table 2). At the same time, leaf pair density showed an initial increase by 24% at 6 PSU after just 1 week, but reduced soon afterwards with significant (p<0.01) reductions at 6 PSU compared to low and mid salinities after 3 weeks. After 6 weeks there were no shoots remaining at 3 PSU and after 10 weeks there were just 3% remaining at 6 PSU. There was a very distinct threshold between 6 and 9 PSU, with leaf pair density increasing relative to starting density and being the highest at salinities ranging from 9 to 15 PSU; however, significant (p<0.01) increases in shoot density occurred only at 12 and 15 PSU. Shoot density increased at 36 and 33 PSU (by 30% and 55%), which was followed by negligible change in density at 30 PSU (2% increase), but density then increased again at lower salinities until reaching mortality thresholds.

**Table 2:** Summary of Tukeys Post-hoc comparisons for each week for change in shoot density.

Week	*H. ovalis*	*H. uninervis*	*Z. muelleri*
1	3<9,12,15,21	n.s.	n.s.
	12>30		
2	3<all others	n.s.	6>15,21–36
3	3<all others	n.s.	3>21–30, 36
	6<9,12,15,21		6>18–36
	12>30,33		9>15–36
			12>18–36
			15>36
4	3<all others	3<9–18	3>15–36
	6<12–24		6>12–36
	12>30		9>15–36
			12>33–36
6	3<6<all others	3<9–36	3<6, 3>21–36
	12>30	6<12,18	6>3, 12–36
			9>15–36
			12>36
8	3 = 6<all others	3<9–36	3<6, 3>21–36
		6<9–18,30–33	6>3, 12–36
			9>15–36
			12>21–30,36
10	3 = 6<all others	3<9–36	3<6, 3>21–36
	15>27–36	6<9–15	6>3, 12–36
			9>15–36
			12>21–30,36

Differences among treatments are indicated for each species at each measuring time

**Figure 4:**
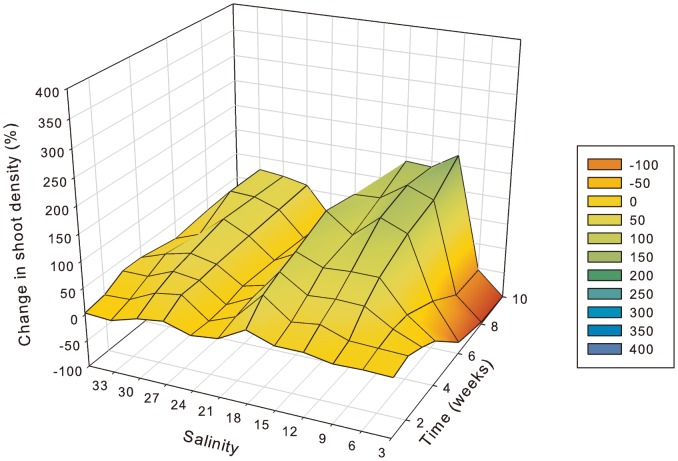
Change in *Halodule uninervis* shoot density relative to pre-treatment (week 0) (y-axis) as indicated by colour shading from 100% loss (red) through to 400% increase (blue), at salinities 3 to 36 PSU (x-axis) after 1 through to 10 weeks of exposure to treatment salinity (z-axis). n = 4.

For *H. uninervis*, the general trends were similar but the reaction time was slower and was more difficult to detect, as initial shoot density was considerably lower. There were no significant differences among treatments up to and including 3 weeks of hypo-salinity exposure (Fig 4). After 4 weeks, density had significantly declined in 3 PSU relative to salinities of 9 through 18 PSU, and after 6 weeks density was significantly lower at 3 PSU than in all other treatments. After 10 weeks, there were no shoots remaining at 3 PSU. At 6 PSU, *H. uninervis* initially increased by 25% after 3 weeks, but started to decline thereafter, being significantly reduced relative to low/mid-range salinities after 4 weeks and there was 54% loss after 10 weeks. There was a distinct threshold between 6 and 9 PSU, with no net loss of shoots at 9 PSU, which instead showed the greatest increase in density among all salinities of 170% after 10 weeks.

**Figure 5:**
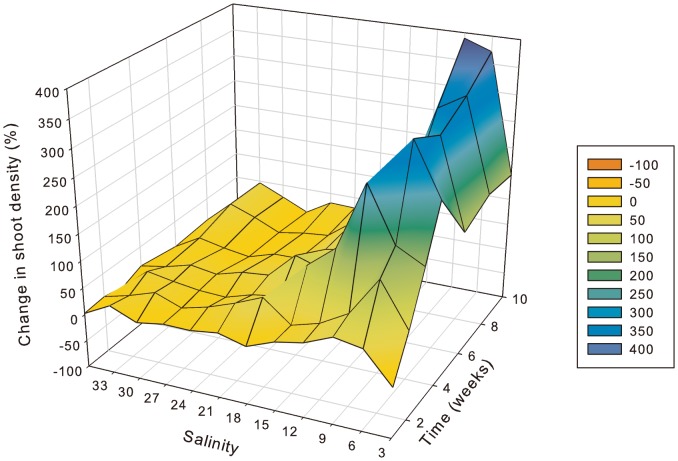
Change in *Zostera muelleri* shoot density relative to pre-treatment (week 0) (y-axis) as indicated by colour shading from 100% loss (red) through to 400% increase (blue), at salinities 3 to 36 PSU (x-axis) after 1 through to 10 weeks of exposure to treatment salinity (z-axis). n = 4.

In *Z. muelleri*, hypo-salinity had a significant and positive effect on shoot density at salinities from 3 to 15 PSU (Fig 5). After 2 weeks, shoot density had increased significantly (p<0.01) more at 6 PSU compared to higher salinities, and after 3 weeks, density had increased significantly (p<0.01) at salinities from 3 to 15 PSU relative to higher salinities. After an initial increase of 240% at 3 PSU within 4 weeks, shoot density started to decline, but remained elevated relative to pre-treatment conditions throughout the experiment and was 150% greater than starting density after 10 weeks. The largest increase in shoot density was at 6 PSU, where density was 400% higher than pre-treatment after 10 weeks. It was significantly (p<0.01) higher at 6 PSU than all other treatments (except 9 PSU) after 4weeks and remained significantly (p<0.01) elevated throughout. At 9 PSU, shoot density was significantly (p<0.01) higher than all salinities of 15 PSU and greater after just 3 weeks. Shoot density was significantly (p<0.01) higher at 12 PSU than at 21–36 PSU (except 33 PSU) after 8 and 10 weeks. The smallest change in shoot density occurred at 36 and 27 PSU, with 0 and 1% increase in shoot density, respectively, after 10 weeks.

### Leaf area

**Figure 6:**
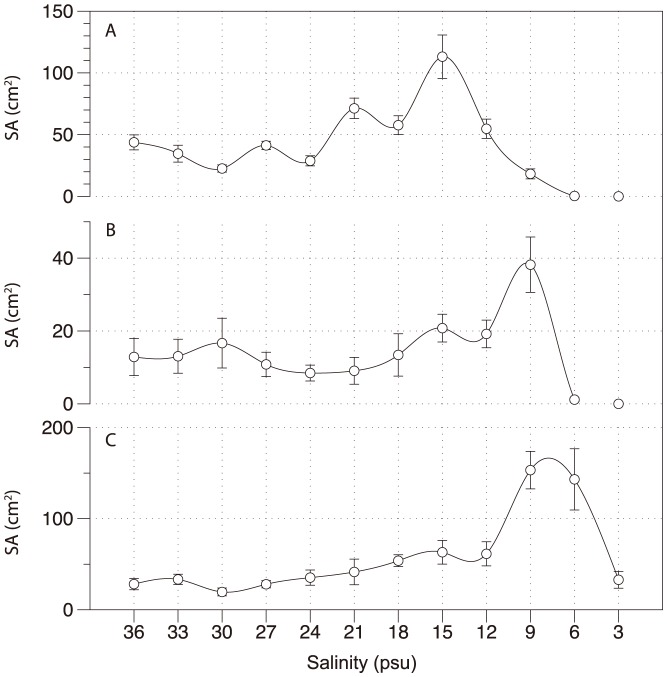
Foliar surface area (SA, cm^2^) calculated from shoot density, leaves per shoot and leaf length and width of *H. ovalis* (A), *H. uninervis* (B) and *Z. muelleri* (C) after 10 weeks at treatment salinity. n = 4 ± SE

Foliar surface area (SA), calculated from shoot density as well as shoot size (leaf length, width and leaves per shoot) after 10 weeks exposure to hypo-salinity treatments, followed the same general trends and magnitude of response as for shoot density. For *H. ovalis*, salinity had a significant effect (F = 42.041, MS = 37.236, p<0.001, SqRt transformed) on SA. The largest SA occurred at 15 PSU, where it was significantly (p<0.01) higher than all other treatments except 21 PSU, and was more than double that at 36 PSU (Fig 6A). The lowest SA occurred at 3 and 6 PSU where there were just 0 and 2 shoots remaining, resulting in a significantly (p<0.001) reduced SA compared to all other treatments. For *H. uninervis* salinity also had a significant effect on SA (MS = 9.989, F = 6.839, p<0.001, SqRt transformation), the peak in SA at 9 PSU was significantly (p<0.01) greater than SA at 3-6 PSU, and 18–24 PSU, inclusive (Fig 6B). SA was significantly (p<0.05) lower at 3 PSU than SA at all salinities except 21 and 24 PSU. For *Z. muelleri*, the significant effect of salinity on SA (MS = 25.711, F = 10.182, p<0.001, SqRt transformation) peaked at 6 and 9 PSU, which were 5 times greater than at 36 PSU, and which were both significantly (p<0.05) higher than all other treatments (Fig 5C).

### Sexual reproduction (flowering)

**Figure 7:**
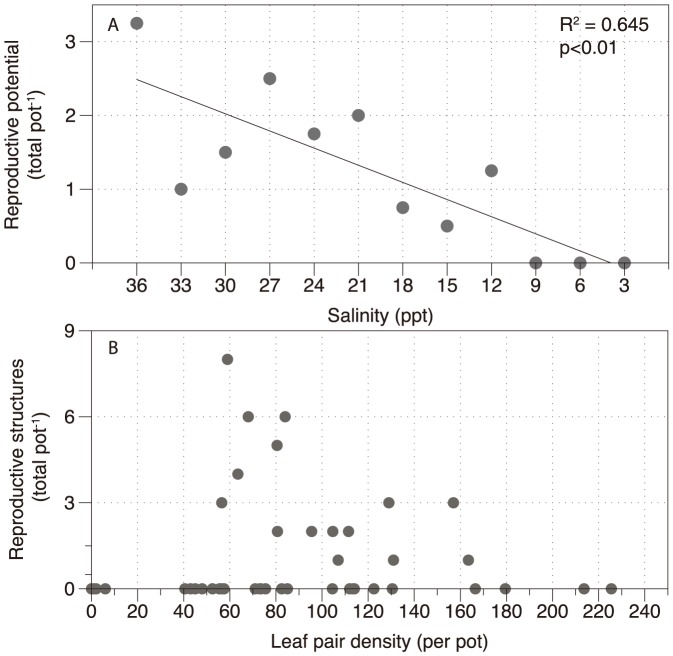
Sexual reproduction in *Halophila ovalis* under salinity treatment conditions showing (A) reproductive potential which is the highest mean (total number of flowers and fruits) recorded for each treatment in weeks 6–10; and, (B) reproductive output (total number of flowers and fruits) correlated with shoot density at 10 weeks. n = 4 ± SE.

Reproductive potential, which is the highest mean recorded in weeks 4–10, increased with salinity, with no structures at 3–9 PSU and the largest number (3.25 pot^−1^) occurring at 36 PSU (Fig 7A). This is in stark contrast to leaf pair density which was greatest at 12–15 PSU for *H. ovalis*. There was an anomaly of reduced reproductive effort at 30 and 33 PSU compared to higher and lower salinities. When plotted against leaf pair density (Fig 7B), the greatest number of reproductive structures occurred at low to moderate leaf pair densities, and at very high leaf pair densities (>165 pairs per pot) there was no reproductive structures, nor at very low densities (<50 pairs), where the plants were generally dying.

### Growth

**Figure 8:**
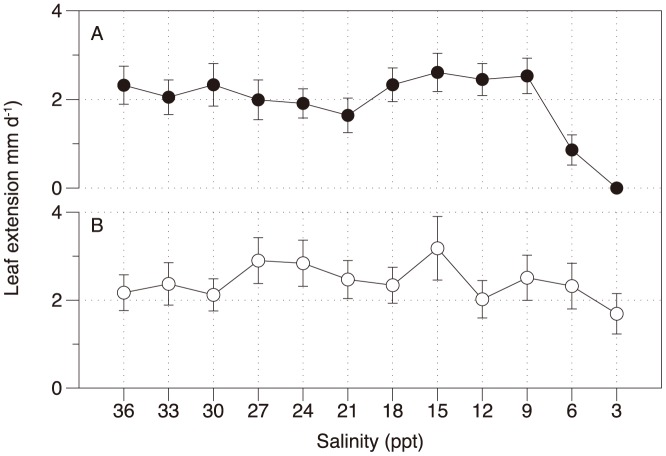
Leaf extension rate (mm d^−1^) for *H. uninervis* (A) and *Z. muelleri* (B) after 10 weeks exposure to hypo-salinity. n = 4 ± SE.

Leaf growth (measured as leaf extension, mm d^−1^) showed very little response to the salinity treatments. *H. uninervis* was significantly affected by the salinity (MS = 2.203, F = 7.955, p<0.001, non-transformed, Fig 8A), but only at 3 and 6 PSU in plants that were essentially dead or almost dead. Growth in 3 PSU was significantly lower than all other treatments, while growth at 6 PSU was significantly lower than 9–18 PSU and 30 PSU and 36 PSU, but not other treatments. Growth in *Z. muelleri* was also significantly affected by salinity (MS = 0.720, F = 2.591 and p<0.05, Fig 8B), with growth at 3 PSU being lower than 15 PSU only.

## Discussion

**Figure 9:**
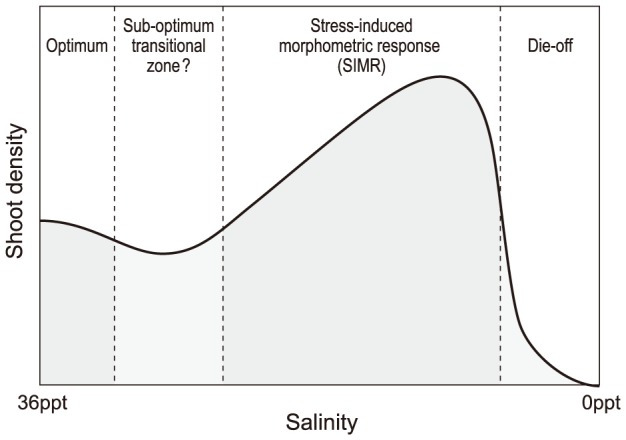
Conceptual summary of the seagrass responses to hypo-salinity. High (marine, 36 PSU) salinities are “optimum”, as shoot density steadily increased throughout the experimental period at this salinity while sexual reproduction (for *H. ovalis*) was at its “peak”. At slightly depressed salinities (30–33 PSU) there appeared to be a “sub-optimal” transition zone as shoot density showed minimal increase and, furthermore, sexual reproduction (for *H. ovalis*) was low. With further hypo-salinity (<30 PSU), a stress-induced morphometric response was associated with a re-prioritisation of resources that saw massively increased shoot density (and leaf area) and reduced sexual reproduction. At extreme hypo-salinity (3–6 PSU) plant mortality occurred. The cut-off for each response phase moved to higher salinities with increased duration of exposure.

These coastal Indo-Pacific seagrasses demonstrated very broad salinity tolerance when gradually exposed to hypo-salinity. Even after 10 weeks exposure, *Zostera muelleri* had survived to salinities as low as 3 PSU, while *Halophila ovalis* and *Halodule uninervis* remained abundant at 9 PSU. However, the plant-scale responses were complex, and the high treatment-resolution enabled us to develop a thorough conceptual model to describe this response (Fig 9). The most distinctive finding was a stress-induced morphometric response (SIMR) [20]–[22], characterised by shoot proliferation. This corresponded to reduced flowering (for *Halophila ovalis*) indicating a diversion of resources away from sexual reproduction to support the lateral branching. This did not come at the expense of leaf growth, which was largely unaffected by salinity, or shoot development (shoot size), as the foliar surface area mirrored the shoot density response. This shoot proliferation was a ‘moderate stress response’ and as the hypo-salinity treatments progressed to lower salinities, severe stress resulted in die-off (Fig 9). In this way, the shoot proliferation preceded mortality.

A ‘sub-optimal transitional zone’ between optimum salinity (36 PSU) and SIMR salinities appeared at 27–33 PSU depending on species, recognizable as a zone with small changes in shoot density (Figs 5 and 6). For example, the smallest change in shoot density occurred at 30 PSU in *H. ovalis* (2%); while at salinities both above (optimum salinity) and below this (stress response) there was shoot proliferation. There was also very low sexual reproduction in this transition zone. Previous studies have reported SIMR responses for other non-seagrass species groups [20]–[22]; however the proposed sub-optimal transitional zone requires further validation.

The broad salinity tolerance indicates intracellular osmoregulation within the plant tissues. In halophytes, selective ion and solute accumulation enables high intra-cellular osmotic potentials to remain. A number of osmolytes occur in seagrass leaves, including, inorganic ions (Na^+^, K^+^, Cl^−^) soluble sugars and amino acids (in particular proline) [3]. Adjusting osmolyte concentration is energetically costly and slow, and this may partially explain why gradual changes in salinity, rather than sudden changes are associated with broad salinity tolerance [3]. Hypo-salinity can progress quickly: for example, sudden changes might result from heavy rainfall falling directly onto very shallow or even exposed intertidal meadows, or during very sudden and heavy run-off. Under these circumstances, the inability to slowly regulate osmolyte concentrations may cause more cellular damage and result in mortality at higher salinities [3], [5], [12].

Threshold salinities associated with mortality were different among species with *H. ovalis* being the most sensitive, and *Z. muelleri* the most tolerant of hypo-salinity. We have compared salinity thresholds associated with sub-lethal and lethal impacts from this study with published findings (Table 3). This comparison focuses on mortality or changes in abundance. Since the studies summarized in the comprehensive review by Touchette [3] there has been considerable research effort exploring salinity stress, in particular physiological responses to hyper-salinity stress in *Thalassia testudinum* (e.g. [5], [17], [28]) and *Posidonia oceanica* (e.g. [18], [29], [30]). As summarized in Table 3 there are fewer data available on hypo-salinity responses, though where measured, seagrasses do tend to have low hypo-salinity thresholds (Table 3). This detailed experimental design has enabled us to identify salinity thresholds with a high level of precision. A significant outcome from this analysis is the identification of a stress-induced morphometric response indicated in Table 3 as a “sub-lethal” response. Furthermore, our exposure time has exceeded that of many previous studies enabling us to consider sensitivity of seagrasses over ‘wet season’ time-scales.

**Table 3:** Summary of the sub-lethal (e.g. growth and photosynthesis or some shoot/biomass loss), and complete mortality thresholds (PSU) for seagrasses in responses to hypo- and hyper-salinity exposure.

					Hypo-	Hypo-	Hyper-	Hyper-		
Species	Experiment	Experimental salinity range	Optimum salinity	Experimental time	Sub-lethal	Mortality	Sub-lethal	Mortality	Sub-lethal measure	Citation
*Amphibolis antarctica*	A	35–65	42	5 mo	-	-	50–57.5	65	Seedling productivity	[33]
*Amphibolis antarctica*	IS	35–58.5	42	8–10 d	-	-	49–60	-	Leaf growth and biomass	[34]
*Cymodocea nodosa*	A	0–72	30–39	10 d	16	0	41	57	Leaf growth	[13]
*Cymodocea nodosa*	A	37–62	37–44	17 d	-	-	54	NE	Leaf growth, PSII efficiency	[35]
*Cymodocea nodosa*	A	37–43	37	47 d	-	-	39	NE	Photosynthesis	[36]
***Halodule uninervis***	**A**	**3**–**36**	**36**	**10 wk**	**15**	**3**	**-**	**-**	**Shoot proliferation, not growth**	**This study**
*Halodule wrightii*	A	35–70	35–65	1 mo	-	-	70	NE	Leaf growth, osmolality, quantum efficiency	[5]
*Halodule wrightii*	A	5–45	35	14 d	5	NE	45	NE	Leaf extension	[4]
*Halophila johnsonii*	A	10–30	20–30	28 d	10	10	-	-	Leaf area, PSII efficiency	[37]
*Halophila johnsonii*	A	8–30	15–30	1 mo	8–10	NE	-	-	PSII efficiency, leaf osmolality, antioxidant activity of leaves	[12]
*Halophila johnsonii*	A	0–60	30	15 d	20	0	40	60	Photosynthesis, growth	[38]
***Halophila ovalis***	**A**	**3**–**36**	**36**	**10 wk**	**12**	**6**	**-**	**-**	**Shoot proliferation, not growth**	**This study**
*Halophila ovalis*	A	5–45	20–35	6 wk	10	5	40	NE	Biomass	[39]
*Posidonia oceanica*	A	25–57	25–39	15 d	NE	29	42	50	Leaf growth	[40]
*Posidonia oceanica*	IS	37–38	37	16 yr	-	-	38–40	NE	Leaf growth, NSC, %N	[7]
*Posidonia oceanica*	A	37–43	37	47 d	-	-	39	NE	Photosynthesis and leaf growth	[41]
*Posidonia oceanica*	A	37–43	37	3 mo	-	-	43	NE	Leaf turgor, photosynthesis, growth	[19]
*Posidonia oceanica*	A	37.5–39	37.5	3 mo	-	-	38.5	NE	Shoot size, leaf growth rate	[30]
*Ruppia cirrhosa*	IS	0–55	0	5 mo	-	-	15	NE	Natural occurrence	[42]
*Ruppia maritima*	A	35–70	35–40	1 mo	-	-	55–70	NE	Leaf growth, shoot density, PSII efficiency	[5]
*Ruppia maritima*	A	0–45	0	150 d	-	-	5	35	Seedling biomass	[31]
*Syringodium filiforme*	A	5–45	25	14 d	5	NE	45	NE	Leaf extension	[4]
*Thalassia testudinum*	A	0–70	30–40	14 wk	10	0	50–60	70	Leaf growth, leaf area, photosynthesis, osmolality	[11]
*Thalassia testudinum*	A	35–65	35–55	2 mo	-	-	65	NE	Leaf growth, shoot density, PSII efficiency	[43]
*Thalassia testudinum*	A	5–45	40	14 d	5	NE	45	NE	Leaf extension	[4]
*Zostera capensis*	IS	0–55	30	5 mo	15	0	-	55	Plant mass	[42]
***Zostera muelleri***	**A**	**3**–**36**	**36**	**10 wk**	**15**	**NE**	**-**	**-**	**Shoot proliferation, not growth**	**This study**

The tested range, time of exposure and the optimum salinity are also given. A  =  Aquarium based, IS  =  *in-situ*, NE  =  No effect, and “-” is not measured. Bold  =  “This study”.

The question remains as to why these species tend to be restricted to waters that are predominantly marine when they are clearly tolerant of hypo-salinity. There are a number of possibilities. Firstly, this study was conducted over a 10-week period to represent a hypo-salinity flood event. Exposure to hypo-salinity for longer than 10 weeks could result in higher mortality rates. Secondly, low salinity events tend to coincide with elevated turbidity and nutrients as well as fast water flows. These other environmental impacts, or potentially synergistic impacts (for example, mortality increased with ammonium concentration in *Thalassia testudinum* [11]) could prevent habitation in brackish, riverine environments, rather than salinity itself. Thirdly, reproductive effort was severely impaired at low salinities – although this could only be measured for *H. ovalis*. In some species (e.g. *Ruppia maritima*), seedling germination is enhanced by rapid osmotic shock from hyper to hypo-salinity [31]; however, this study demonstrates that seed production, in these species was inhibited by chronic exposure to hypo-salinity. *Halophila ovalis* is a colonizing species, which is highly dependent on seed production for long-term survival and disruptions to sexual reproduction would probably prevent population survival. Furthermore, if seed production and germination are successful, seedling development is highly sensitive to small changes in salinity [32].

In conclusion, hypo-salinity stress caused a stress-induced morphometric response (SIMR) followed by severe mortality in *H. ovalis* and *H. uninervis* at salinities less than 9 PSU. If observed in natural conditions, a SIMR could suggest that the population is not only healthy, but is in fact in a trajectory of increasing abundance when using traditional monitoring tools, such as shoot density or percent cover. A critical next step is to explore how other interacting factors can affect responses to hypo-salinity.

## References

[1] Les DH, Cleland MA, Waycott M (1997) Phylogenetic studies in Alismatidae, II: Evolution of marine angiosperms (seagrasses) and hydrophily. Syst Bot 22: 443–463.

[2] Janssen T, Bremer K (2004) The age of major monocot groups inferred from 800+rbcL sequences. Bot J Linn Soc 146: 385–398.

[3] Touchette BW (2007) Seagrass-salinity interactions: Physiological mechanisms used by submersed marine angiosperms for a life at sea. J Exp Mar Biol Ecol. 350: 194–215.

[4] Lirman D, Cropper WP Jr (2003) The influence of salinity on seagrass growth, survivorship, and distribution within Biscayne Bay, Florida: field, experimental, and modeling studies. Estuaries 26: 131–141.

[5] Koch MS, Schopmeyer SA, Kyhn-Hansen C, Madden CJ, Peters JS (2007) Tropical seagrass species tolerance to hypersalinity stress. Aquat Bot. 86: 14–24.

[6] Walker DI, Kendrick GA, McComb AJ (1988) The distribution of seagrass species in shark bay, Western Australia, with notes on their ecology. Aquat Bot. 30: 305–317.

[7] Gacia E, Invers O, Manzanera M, Ballesteros E, Romero J (2007) Impact of the brine from a desalination plant on a shallow seagrass (*Posidonia oceanica*) meadow. Estuarine, Coastal and Shelf Science 72: 579–590.

[8] Furnas M (2003) Catchment and corals: terrestrial runoff to the Great Barrier Reef. Townville Queensland: Australian Institute of Marine Science. 334 p.

[9] Preen AR, Long WJL, Coles RG (1995) Flood and cyclone related loss, and partial recovery, of more than 1000 km^2^ of seagrass in Hervey Bay, Queensland, Australia. Aquat Bot. 52: 3–17.

[10] Campbell SJ, McKenzie LJ (2004) Flood related loss and recovery of intertidal seagrass meadows in southern Queensland, Australia. Estuarine, Coastal and Shelf Science 60: 477–490.

[11] Kahn AE, Durako MJ (2006) *Thalassia testudinum* seedling responses to changes in salinity and nitrogen levels. J Exp Mar Biol Ecol. 335: 1–12.

[12] Griffin NE, Durako MJ (2012) The effect of pulsed versus gradual salinity reduction on the physiology and survival of *Halophila johnsonii* Eiseman. Marine Biology 159: 1439–1447.

[13] Fernandez-Torquemada Y, Sanchez-Lizaso JL (2011) Responses of two Meditteranean seagrasses to experimental changes in salinity. Hydrobiologia 669: 21–33.

[14] Chaves MM, Flexas J, Pinheiro C (2009) Photosynthesis under drought and salt stress: regulation mechanisms from whole plant to cell. Annals of Botany 103: 551–560. 18662937 10.1093/aob/mcn125 PMC2707345

[15] Munns R, Tester M (2008) Mechanisms of salinity tolerance. Annu Rev Plant Biol 59: 651–681. 18444910 10.1146/annurev.arplant.59.032607.092911

[16] Ralph P (1998) Photosynthetic responses of *Halophila ovalis* (R. Br.) Hook. *f*. to osmotic stress. J Exp Mar Biol Ecol. 227: 203–220.

[17] Howarth JF, Durako MJ (2013) Variation in pigment content of *Thalssia testudinum* seedlings in response to changes in salinity and light. Bot Mar. 56: 261–273.

[18] Sandoval-Gil J, Marín-Guirao L, Ruiz J (2012) Tolerance of Mediterranean seagrasses (*Posidonia oceanica* and *Cymodocea nodosa*) to hypersaline stress: water relations and osmolyte concentrations. Marine Biology 159: 1129–1141.

[19] Marín-Guirao L, Sandoval-Gil JM, Bernardeau-Esteller J, Ruíz JM, Sánchez-Lizaso JL (2013) Responses of the Mediterranean seagrass *Posidonia oceanica* to hypersaline stress duration and recovery. Mar Environ Res. 84: 60–75. 23306019 10.1016/j.marenvres.2012.12.001

[20] Potters G, Pasternak TP, Guisez Y, Palme KJ, Jansen MAK (2007) Stress-induced morphogenic responses: growing out of trouble? Trends Plant Sci. 12: 98–105. 17287141 10.1016/j.tplants.2007.01.004

[21] Potters G, Pasternak TP, Guisez Y, Jansen MAK (2009) Different stresses, similar morphogenic responses: integrating a plethora of pathways. Plant, Cell & Environment 32: 158–169. 10.1111/j.1365-3040.2008.01908.x 19021890

[22] Zolla G, Heimer YM, Barak S (2010) Mild salinity stimulates a stress-induced morphogenic response in *Arabidopsis thaliana* roots. J Exp Bot. 61: 211–224. 19783843 10.1093/jxb/erp290 PMC2791118

[23] Waycott M, McMahon K, Mellors J, Calladine A, Kleine D (2004) A guide to tropical seagrasses of the Indo-West Pacific. Townsville: James Cook University. 72 p.

[24] Collier CJ, Waycott M, Giraldo-Ospina A (2012) Responses of four Indo-West Pacific seagrass species to shading. Mar Pollut Bull 65: 342–354. 21741666 10.1016/j.marpolbul.2011.06.017

[25] McMahon KM (2005) Recovery of subtropical seagrasses from natural disturbance. Brisbane: The University of Queensland. 198 p.

[26] Short FT, Duarte CM (2001) Methods for the measurement of seagrass growth and production. In: Short FT, Coles R, editors. Global seagrass research methods. Amsterdam: Elsevier Science. pp. 473.

[27] Underwood AJ (1997) Experiments in ecology: Their logical design and interpretation using analysis of variance. Cambridge: Cambridge University Press.

[28] Jiang Z, Huang X, Zhang J (2013) Effect of nitrate enrichment and salinity reduction on the seagrass *Thalassia hemprichii* previously grown in low light. J Exp Mar Biol Ecol. 443: 114–122.

[29] Marín-Guirao L, Ruiz JM, Sandoval-Gil JM, Bernardeau-Esteller J, Stinco CM, et al (2013) Xanthophyll cycle-related photoprotective mechanism in the Mediterranean seagrasses *Posidonia oceanica* and *Cymodocea nodosa* under normal and stressful hypersaline conditions. Aquat Bot. 109: 14–24.

[30] Ruiz JM, Marin-Guirao L, Sandoval-Gil JM (2009) Responses of the Mediterranean seagrass *Posidonia oceanica* to *in situ* simulated salinity increase. Bot Mar. 52: 459–470.

[31] Strazisar T, Koch MS, Madden CJ, Filina J, Lara PU, et al (2013) Salinity effects on *Ruppia maritima* L. seed germination and seedling survival at the Everglades-Florida Bay ecotone. J Exp Mar Biol Ecol. 445: 129–139.

[32] Kirkman H, Kuo J (1990) Pattern and process in southern Western Australian seagrasses. Aquatic Botany 37: 367–382.

[33] Walker DI, McComb AJ (1990) Salinity response of the seagrass *Amphibolis antarctica* (Labill.) Sonder et Aschers.: an experimental validation of field results. Aquat Bot. 36: 359–366.

[34] Walker DI (1985) Correlations between salinity and growth of the seagrass *Amphibolis antarctica* (labill.) Sonder ex Aschers., In Shark Bay, Western Australia, using a new method for measuring production rate. Aquat Bot. 23: 13–26.

[35] Pages JF, Perez M, Romero J (2010) Sensitivity fo the seagrass *Cymodocea nodosa* to hypersaline conditions: a microcosm approach. J Exp Mar Biol Ecol. 386: 34–38.

[36] Sandoval-Gil JM, Marín-Guirao L, Ruiz JM (2012) The effect of salinity increase on the photosynthesis, growth and survival of the Mediterranean seagrass *Cymodocea nodosa* . Estuarine, Coastal and Shelf Science 115: 260–271.

[37] Kahn A, Durako MJ (2008) Photophysiological responses of *Halophila johnsonii* to experimental hyposaline and hyper-CDOM conditions. J Exp Mar Biol Ecol. 367: 230–235.

[38] Torquemada YF, Durako MJ, Lizaso JLS (2005) Effects of salinity and possible interactions with temperature and pH on growth and photosynthesis of *Halophila johnsonii* . Eiseman. Marine Biology 148: 251–260.

[39] Hillman K, McComb AJ, Walker DI (1995) The distribution, biomass and primary production of the seagrass *Halophila ovalis* in the Swan/Canning Estuary, Western Australia. Aquat Bot. 51: 1–54.

[40] Fernández-Torquemada Y, Sánchez-Lizaso JL (2005) Effects of salinity on leaf growth and survival of the Mediterranean seagrass *Posidonia oceanica* (L.) Delile. J Exp Mar Biol Ecol. 320: 57–63.

[41] Marín-Guirao L, Sandoval-Gil JM, Ruíz JM, Sánchez-Lizaso JL (2011) Photosynthesis, growth and survival of the Mediterranean seagrass *Posidonia oceanica* in response to simulated salinity increases in a laboratory mesocosm system. Estuarine, Coastal and Shelf Science 92: 286–296.

[42] Adams JB, Bate GC (1994) The tolerance to desiccation of the submerged macrophytes *Ruppia cirrhosa* (Petagna) grande and *Zostera capensis* setchell. J Exp Mar Biol Ecol. 183: 53–62.

[43] Koch MS, Schopmeyer SA, Holmer M, Madden CJ, Kyhn-Hansen C (2007) *Thalassia testudinum* response to the interactive stressors hypersalinity, sulfide and hypoxia. Aquat Bot. 87: 104–110.

